# Neurofilaments as a plasma biomarker for ICU-acquired weakness: an observational pilot study

**DOI:** 10.1186/cc13699

**Published:** 2014-01-20

**Authors:** Luuk Wieske, Esther Witteveen, Axel Petzold, Camiel Verhamme, Marcus J Schultz, Ivo N van Schaik, Janneke Horn

**Affiliations:** Department of Intensive Care Medicine, Academic Medical Center, University of Amsterdam, Meibergdreef 9, 1105 AZ Amsterdam, the Netherlands; Department of Neurology, Academic Medical Center, Amsterdam, the Netherlands; Laboratory of Experimental Intensive Care and Anesthesiology (L·E·I·C·A), Academic Medical Center, Amsterdam, the Netherlands; Department of Neurology, VU medisch centrum Amsterdam, Amsterdam, the Netherlands; Department of Neuroimmunology, UCL Institute of Neurology, Queen Square, London, UK

## Abstract

**Introduction:**

Early diagnosis of intensive care unit – acquired weakness (ICU-AW) using the current reference standard, that is, assessment of muscle strength, is often hampered due to impaired consciousness. Biological markers could solve this problem but have been scarcely investigated. We hypothesized that plasma levels of neurofilaments are elevated in ICU-AW and can diagnose ICU-AW before muscle strength assessment is possible.

**Methods:**

For this prospective observational cohort study, neurofilament levels were measured using ELISA (NfH^SMI35^ antibody) in daily plasma samples (index test). When patients were awake and attentive, ICU-AW was diagnosed using the Medical Research Council scale (reference standard). Differences and discriminative power (using the area under the receiver operating characteristic curve; AUC) of highest and cumulative (calculated using the area under the neurofilament curve) neurofilament levels were investigated in relation to the moment of muscle strength assessment for each patient.

**Results:**

Both the index test and reference standard were available for 77 ICU patients. A total of 18 patients (23%) fulfilled the clinical criteria for ICU-AW. Peak neurofilament levels were higher in patients with ICU-AW and had good discriminative power (AUC: 0.85; 95% CI: 0.72 to 0.97). However, neurofilament levels did not peak before muscle strength assessment was possible. Highest or cumulative neurofilament levels measured before muscle strength assessment could not diagnose ICU-AW (AUC 0.59; 95% CI 0.37 to 0.80 and AUC 0.57; 95% CI 0.32 to 0.81, respectively).

**Conclusions:**

Plasma neurofilament levels are raised in ICU-AW and may serve as a biological marker for ICU-AW. However, our study suggests that an early diagnosis of ICU-AW, before muscle strength assessment, is not possible using neurofilament levels in plasma.

**Electronic supplementary material:**

The online version of this article (doi:10.1186/cc13699) contains supplementary material, which is available to authorized users.

## Introduction

Intensive Care Unit–acquired weakness (ICU-AW) is a frequent neuromuscular complication of critical illness [1]. The disorders causing ICU-AW are critical illness myopathy (CIM), critical illness polyneuropathy (CIP) or critical illness neuromyopathy (CINM) [2]. ICU-AW has important implications for critically ill patients because mortality and (long-term) morbidity are increased in ICU-AW [1]. The current standard for diagnosing ICU-AW is muscle strength assessment, quantified using the Medical Research Council (MRC) scale [2]. In critically ill patients, muscle strength assessment is frequently delayed because of impaired consciousness or attentiveness, due to sedation or delirium [3]. It is important to diagnose ICU-AW early after onset of critical illness to initiate supportive interventions, such as early rehabilitation, which may improve functional outcome [4]. Additionally, it is important to provide accurate prognostic information to patients, their families and physicians.

Biomarkers for ICU-AW may facilitate an early diagnosis. So far, biomarkers of ICU-AW have been scarcely investigated. Creatine kinase, a marker for muscle damage, is probably not an accurate diagnostic marker for ICU-AW [2]. Neurofilaments are a biomarker for axonal injury and, in recent years, have been investigated in various neurological conditions, such as Guillain-Barré syndrome [5]. CINM and CIP both cause axonal injury and neurofilaments might, therefore, be a biomarker for ICU-AW [2].

The purpose of this study was to investigate the diagnostic accuracy of neurofilament levels in plasma as an early marker for ICU-AW. We hypothesized that: 1) plasma levels of neurofilaments are elevated in patients with ICU-AW; and 2) plasma levels of neurofilaments can diagnosis ICU-AW early, before muscle strength assessment is possible.

## Methods

### Design and ethical approval

We conducted a prospective observational cohort study in the mixed medical-surgical ICU of the Academic Medical Center, Amsterdam, the Netherlands. This manuscript was drafted in accordance with the Standards for the Reporting of Diagnostic accuracy studies STARD criteria [6]. The Institutional Review Board approved the study (10/219 # 10.17.1630) and waived the need for informed consent, because leftover plasma of blood samples obtained for routine care was used for this study.

### Inclusion and exclusion criteria

Newly admitted ICU patients were eligible for inclusion in this study, except patients who were admitted because of stroke, traumatic brain or spinal injury, a neuromuscular disorder or an infection of the central nervous system. In addition, we excluded patients after cardiac arrest and patients who were admitted for post–operative observation after elective surgery or emergency cardiothoracic surgery. Finally, we excluded patients with a poor pre-hospital functional status (modified Rankin >3 [7]) and patients with pre-existing spinal injury or neurodegenerative disorders.

### Medical Research Council scale for muscle strength (reference standard)

Physical therapists performed manual muscle strength testing as soon as patients were awake (defined as Richmond Sedation and Agitation Scale (RASS) between −1 (‘drowsy’) and 1 (‘restless’)) and attentive (able to adequately respond to verbal commands with arms or eyelids). This was done as part of routine care. Factors precluding strength assessment, such as delirium or sedation, were also noted. Muscle strength examination using the MRC scale has good intra- and inter-observer variability, when employed in awake and attentive patients [3, 8, 9]. Muscle strength was assessed in a protocolized way using the MRC scale and scored as an integer. The following muscle groups were tested bilaterally: shoulder abductors, elbow flexors, wrist flexors, hip flexors, knee extensors and ankle dorsiflexors. The assessment was done without knowing neurofilament results. Individual muscle scores were summated and divided by the number of muscles tested to obtain an average MRC score. ICU-AW was defined as an average MRC-score <4 [2].

### Collection of samples and measurement of neurofilaments (index test)

For every day of ICU admission, samples were collected from leftover ethylenediaminetetraacetic acid (EDTA) plasma used for routine care. Plasma was aliquoted with a sampling code blinded for study outcome and stored at −80°C within four hours after they were obtained from patients.

Neurofilament levels were measured using a previously described ELISA [5, 10]. After all samples had been collected, analyses were done batch-wise, to minimize differences in standard preparation and buffers [11]. Samples containing less than 100 μl were measured once; all other samples were measured in duplicate. As a capture antibody we used NfH^SMI35^ (SMI-35R; Covance Inc, Princeton NJ, USA), which binds various phosphorylated forms of the heavy-chain neurofilament protein [5]. The median analytical error (coefficient of variation; CV) was 7.9% (interquartile range (IQR): 3.7 to 14.0). For samples with CV values >30%, the ELISA was repeated, if sufficient plasma was still available. Samples with CV values >30% at re-measurement or samples that could not be re-measured were excluded from the analysis. For samples with neurofilament levels <1 ng/ml a CV >30% was accepted. The detection limit in this study was 0.026 ng/ml. For samples with a neurofilament level below the detection limit we imputed the neurofilament level at half the detection limit (that is, 0.013 ng/ml). Sample analysis and selection was performed blinded to study outcome.

### Clinical data collection

The following clinical characteristics were collected: age, gender, admission type, presence of sepsis, severe sepsis and septic shock during admission (definitions according to [12] and [13], respectively), Acute Physiology and Chronic Health Evaluation IV (APACHE IV) score, presence of multiple organ dysfunction syndrome (MODS) during admission (defined as organ failure (organ specific Sequential Organ Failure Assessment (SOFA) score >2) in two or more organ-systems occurring on the same day [14]) and presence of central nervous system (CNS) organ failure during admission (defined as CNS SOFA score >2 on any day during admission). In addition, data on neurological co-morbidities were collected: presence of risk factors for polyneuropathy (such as diabetes mellitus, alcohol abuse, renal insufficiency or chemotherapy), presence of a pre-existing polyneuropathy and presence of CNS disorders in the medical history (such as stroke or epilepsy).

### Statistical analysis

Depending on the distribution, data are presented as mean values with standard deviation (± SD), as median values with IQR or as proportions with percentages and total numbers. Differences between proportions were assessed using Fisher’s exact test. Differences between normally distributed variables were assessed using Welch’s t-test; differences between non-normally distributed continuous variables were assessed using the Wilcoxon rank-sum test.

To investigate if neurofilament levels are elevated in ICU-AW, we assessed differences in neurofilament peak levels occurring any time during admission. Discriminative power was analyzed by constructing receiver operating characteristic (ROC) curves. From ROC curves, the area under the curve (AUC) with 95% confidence interval (CI) was calculated. We defined discriminative power of AUC values between 0.90 to 1 as excellent, between 0.80 to 0.90 as good, between 0.70 to 0.80 as fair, between 0.60 to 0.70 as poor and <0.60 as failed. The optimal cut-off point of the ROC curve was defined as the point with maximal distance from the diagonal reference line and the sensitivity and specificity of that cut-off point were calculated. Differences between the day of neurofilament peak levels and day of muscle strength assessment were analyzed using the paired samples Wilcoxon signed-rank test.

We also investigated if neurofilaments could diagnose ICU-AW early, that is, before muscle strength assessment was possible. To adjust for the differences in ICU length of stay, we investigated this in a cohort of equal observation length. To define ‘early’ we chose the day before which, in 50% of the patients, muscle strength could have been assessed. Patients in whom strength could not be assessed before this day were used in this analysis. Samples from this group obtained before this 50th percentile day were used to analyze differences in, and discriminative power of, the highest and cumulative neurofilament levels. Cumulative levels were calculated using the area under the neurofilament curve of individual patients.

Finally, we investigated the influences of chronic neurological co-morbidities on admission and peak neurofilament levels occurring at any time during admission. Admission neurofilament levels were defined as samples from ICU day 0 or 1. Also, we investigated the influence of concomitant critical illness-induced CNS organ failure on peak neurofilament levels occurring any time during admission.

Statistical significance is defined as *P* ≤0.05. Analyses were done using R (version: 2.15.2; R Foundation for Statistical Computing, Vienna, Austria). ROC analyses were done using the pROC package [15]. This cohort study was an observational exploratory study and in view of the explorative nature of this study the sample size was chosen on pragmatic grounds. Moreover, no previous data on plasma neurofilament levels in patients with ICU-AW exist, making it impossible to perform a reliable power calculation.

## Results

**Figure 1:**
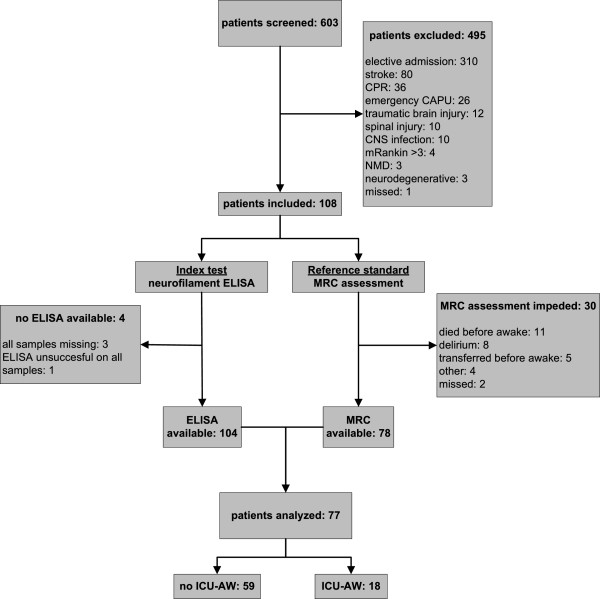
**Flow chart describing enrollment of patients.** CAPU, cardiopulmonary surgery; CNS, central nervous system; CPR, cardiopulmonary resuscitation; ICU-AW, Intensive Care Unit–acquired weakness; MRC, muscle strength as assessed with the Medical Research Council scale; NMD, neuromuscular disorder.

Between November 2011 and May 2012, a total of 108 patients were included in the study (Figure 1). In 77 patients both muscle strength assessment (reference standard) and results from the neurofilament ELISA (index test) were available. Unless otherwise specified, this population was used for analysis. Patient characteristics are displayed in Table 1. From these 77 patients, 618 plasma samples were collected over time. The ELISA was successfully performed in 583 of 618 (94%) samples; reasons for unsuccessful ELISA were a CV of >30% in 13 samples and not enough plasma in 22 samples. A total of 534 of 583 (92%) samples were measured in duplicate; the remainder were measured once.

**Table 1:** **Patient characteristics**

		ICU-AW (N:18)	no ICU-AW (N:59)	***P*** -values
**Demographics**				
Men, number (%)		10 (56)	38 (64)	0.58
Age, mean year ± SD		64 ± 15	60 ± 15	0.25
**Neurological co-morbidities**				
Risk factors for polyneuropathy, number (%)		8 (44)	28 (47)	1
Pre-existing polyneuropathy, number (%)		2 (11)	2 (3)	0.23
History of CNS disorder, number (%)		3 (17)	5 (8)	0.38
Any neurological co-morbidity, number (%)^a^		12 (67)	34 (57)	0.59
**Admission characteristics**				
Admission type				
Medical, number (%)		12 (67)	45 (76)	0.54
Surgical, number (%)		6 (33)	14 (24)	
LOS ICU, median days (IQR)		15 (6 to 34)	3 (1 to 6)	<0.01
APACHE IV score, mean ± SD		85 ± 25	68 ± 22	0.02
Sepsis during admission, number (%)		18 (100)	45 (76)	0.03
Severe sepsis during admission, number (%)		17 (94)	30 (51)	<0.01
Septic shock during admission, number (%)		14 (78)	15 (25)	<0.01
MODS during admission, number (%)		16 (89)	30 (51)	0.01
CNS organ failure during admission, number (%)		8 (44)	4 (7)	<0.01
Moment of muscle strength assessment, median day (IQR)		8 (5 to 14)	4 (2 to 6)	<0.01
Average MRC score, median (IQR)		2.8 (1.7 to 3.6)	4.8 (4.2 to 5)	

^a^Presence of at least one of the individual neurological co-morbidities within a patient. APACHE IV, Acute Physiology and Chronic Health Evaluation IV score; CNS, central nervous system; ICU-AW, Intensive Care Unit–acquired weakness; LOS ICU, length of stay in the intensive care unit; MODS, Multiple Organ Dysfunction syndrome.

### Neurofilaments levels in ICU-AW

**Figure 2:**
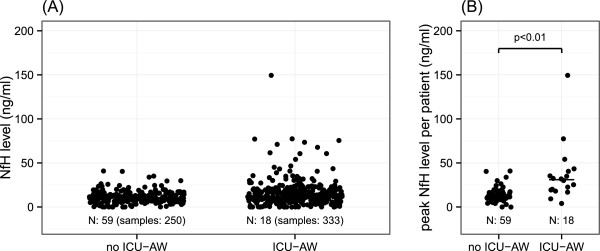
**Neurofilaments in patients with and without Intensive Care Unit-acquired weakness.** Neurofilament levels in all plasma samples **(A)** and peak **(B)** neurofilament levels per patients. Horizontal bars show median group values. ICU-AW, Intensive Care Unit–acquired weakness; MRC, Medical Research Council; NfH, neurofilaments.

**Figure 3:**
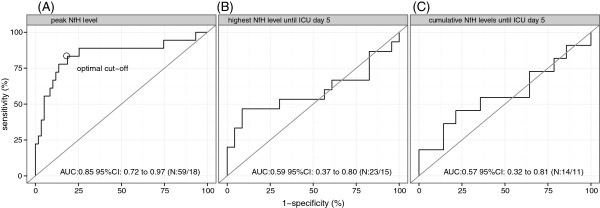
**Neurofilament receiver operating characteristic curves for Intensive Care Unit–acquired weakness.** Overview of discriminative power of different neurofilament measures. **A)** displays peak neurofilament levels at any time during admission. **B)** displays the highest neurofilament level and **C)** cumulative neurofilament level before muscle strength assessment was possible, in a diagnostically relevant cohort of patients (see text for explanation). The number of patients analyzed per curve is denoted as N: number of patients without ICU-AW/number of patients with ICU-AW. Some additional patients are excluded in **C)** when compared to **B)**, because both admission and ICU day 4 samples needed to be present to calculate cumulative neurofilament levels (that is, area under the neurofilament curve). AUC, area under the receiver operating characteristic curve; MRC, Medical Research Council; NfH, neurofilaments.

**Figure 4:**
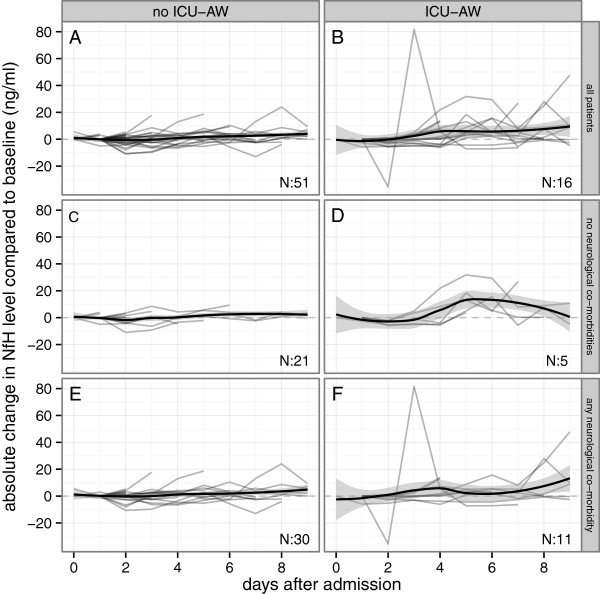
**Profile of plasma neurofilament levels in patients with and without Intensive Care Unit-acquired weakness.** Longitudinal neurofilament profiles, represented as changes compared to baseline levels, over a 10 day period following admission to ICU. Light gray lines represent individual patients; the black lines summarize group levels using local fit regression lines (with the 95% confidence curves in the grey shaded area). In patients without ICU-AW, neurofilament levels remained relatively stable on a group level **(A)**, regardless of the presence of chronic neurological co-morbidities **(C and E)**. In patients with ICU-AW there was a rise over time **(B)**. ICU-AW patients without any chronic neurological co-morbidity had a more homogenous neurofilament profile **(D)** when compared to patients with any chronic neurological co-morbidity **(F)**. Data are missing for seven patients because admission blood samples were not obtained or could not be analyzed successfully. ICU-AW, Intensive Care Unit–acquired weakness; NfH, neurofilaments.

Figure 2A shows neurofilament levels in all plasma samples for patients with and without ICU-AW. Additional file 1: Figure S1 shows plasma levels of patients in whom muscle strength assessment was not possible. Peak neurofilament levels occurring at any time during ICU admission were higher for patients with ICU-AW (Figure 2B). Discriminative power of peak neurofilament levels was good as shown in Figure 3A. The optimal cut-off was 17.9 ng/ml (sensitivity: 83% and specificity: 81%). Neurofilament levels of patients with ICU-AW peaked at ICU day 7 (median, IQR: 5 to 14). This was not significantly earlier than the moment of muscle strength assessment (Table 1; *P* 0.96). Figure 4 shows a longitudinal profile of neurofilament levels.

### Early diagnosis of ICU-AW before muscle strength assessment

Before ICU day 5, 50% of all patients could be assessed for muscle strength. This yielded a total of 38 patients for the subgroup analysis of early diagnosis (15 with ICU-AW and 23 patients without ICU-AW). In this group, highest neurofilament levels until ICU day 5 did not differ between ICU-AW or no ICU-AW (median: 10.7 ng/ml (IQR: 7.2 to 20.6) versus 11.7 ng/ml (IQR: 10.5 to 13.5); *P* 0.37), nor did the cumulative levels until ICU day 5 (median: 26.9 ng/ml (IQR: 16.6 to 41.5) versus 28.8 ng/ml (IQR: 25.5 to 37.0) ICU-AW; *P* 0.61). Discriminative power was not significant (Figure 3B and C).

### Influences of concomitant CNS organ failure and chronic neurological co-morbidities on neurofilament levels

**Figure 5:**
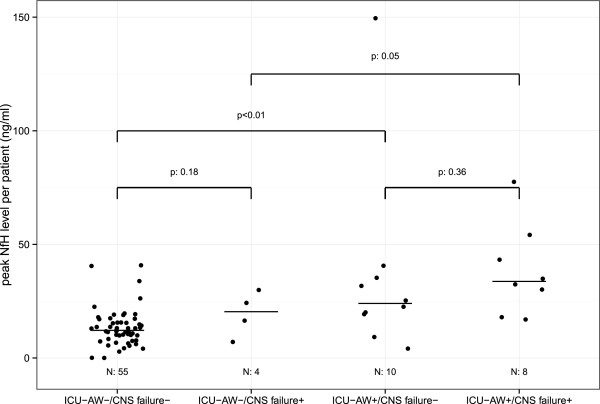
**Peak neurofilament levels and concomitant central nervous system organ failure.** Peak neurofilaments for patients with and without Intensive Care Unit–acquired weakness (ICU-AW) and with and without central nervous system (CNS) failure. Horizontal black bars show median group values. Overall test for differences between groups (that is, Kruskal-Wallis), *P* <0.01; because of the exploratory nature there was no adjustment for multiple comparisons. CNS, central nervous system; ICU-AW, Intensive Care Unit–acquired weakness; NfH, neurofilaments.

In Figure 5 peak neurofilament levels occurring at any time during ICU admission are shown for patients with and without ICU-AW and with and without CNS organ failure. Higher peak neurofilament levels were observed in patients with ICU-AW when compared to patients without ICU-AW, both when CNS organ failure was not present (*P* <0.01) and when CNS organ failure was present (*P* 0.05).

Admission neurofilament levels were not different for patients with any of the chronic neurological co-morbidities (Additional file 2: Figure S2a). Peak neurofilament levels observed at any time during admission were higher for patients with a pre-existing polyneuropathy compared to patients without a pre-existing polyneuropathy; no differences in peak levels were observed for any of the other chronic neurological co-morbidities (Additional file 2: Figure S2b).

## Discussion

This study shows that neurofilament levels are elevated in patients with ICU-AW compared to patients without ICU-AW. Peak neurofilament levels have good discriminative power, but peak levels were not observed prior to the moment of muscle strength assessment. Neurofilament levels could not diagnose ICU-AW before muscle strength assessment. This suggests that neurofilaments are not useful as an early diagnostic marker for ICU-AW.

### Neurofilaments as an early marker for ICU-AW

Our study is the first to measure neurofilaments in plasma as a biological marker for ICU-AW. Neurofilaments are markers of structural damage to axons, and raised neurofilaments in patients with ICU-AW would suggest structural damage to peripheral nerves. In contrast to our hypothesis, neurofilaments did not allow for an early diagnosis of ICU-AW, because neurofilament levels did not peak before muscle strength assessment was possible. This supports the theory that in ICU-AW, structural damage of peripheral nerves develops after a period of dysfunction. Therefore, damage is a late event [16]. Our results do suggest that structural damage occurs much earlier than reported in previous studies, which found histopathological signs of structural damage only weeks after the onset of critical illness [16].

### Other etiologies for raised neurofilaments in critically ill patients

Neurofilaments are non-specific markers for axonal injury. They have been identified as possible markers, either in blood or cerebrospinal fluid, for several peripheral and central neurological disorders, such as Guillain-Barré syndrome, stroke, multiple sclerosis, amyotrophic lateral sclerosis, spinal cord injury and post-anoxic coma [5, 17–21]. Therefore, raised neurofilament levels in patients with ICU-AW may also have another origin. Concomitant critical illness-induced CNS injury may contribute to systemic neurofilament levels. Critical illness can induce axonal damage in the CNS [22]. ICU-AW and CNS organ failure are both manifestations of MODS and will frequently co-occur in patients [23, 24]. In our study we found two patients with serious CNS organ failure and exceptionally high neurofilament levels. We did not find differences in peak neurofilament levels for patients with or without CNS organ failure when compared to patients with or without ICU-AW. However, this may be caused by small subgroup sizes.

Another source for systemic neurofilament levels may be pre-existing neurological disorders [21]. Except for patients with a pre-existing polyneuropathy, we did not find differences in neurofilament peak or admission levels for any of the chronic neurological co-morbidities. Longitudinal neurofilament profiles suggest different patterns for patients with and without chronic neurological co-morbidities. However, group sizes were too small and heterogeneous to draw a reliable conclusion.

### Other modalities for an early diagnosis of ICU-AW

Other diagnostic modalities may also be valuable to establish an early diagnosis of ICU-AW. Early measurement of muscle excitability using direct muscle stimulation, a specialized form of electrophysiological studies, has been found to reliably predict development of ICU-AW [25]. The technique of direct muscle stimulation is not widely available and electrophysiological studies in general are difficult to perform in the ICU setting because of technical limitations, such as limb edema or electrical interference. Another promising diagnostic modality is early muscle ultrasound [26]. Sensitivity and specificity for prediction of a clinical diagnosis of ICU-AW are not yet known.

### Limitations

In this first exploratory study, we used the current reference standard to diagnose ICU-AW, as proposed by international guidelines [2]. Muscle strength assessment cannot discriminate between CINM, CIP and CIM as the underlying disorder for ICU-AW [2]. Although CINM is thought to be most prevalent, we cannot exclude the possibility that some of our patients, in fact, had isolated CIM as the underlying disorder causing ICU-AW [2].

Second, our study design may have led to information bias. Patients without ICU-AW had a shorter ICU stay and muscle strength could be assessed earlier. In this group fewer serum samples were collected. This may have led to an overestimation of the observed differences. For our analysis of neurofilaments as an early diagnostic marker, we corrected for this possible bias by selecting a cohort of equal observation length.

Third, because we were unable to perform a reliable power analysis, there is a risk that our study is underpowered. Finally, we only investigated neurofilaments recognized by the NfH^SMI35^ antibody. NfH^SMI35^ has the best technical characteristics of all neurofilament heavy chain antibodies, but it does not bind to all different phosphorylated forms [5]. In axonal injury, different degrees of phosphorylation have been described and other neurofilament heavy chain phosphoforms or a combination with other neurofilament isoforms, such as the light chain, might provide better diagnostic accuracy in ICU-AW [17].

### Recommendations for future studies

Although probably not suitable as an early marker for ICU-AW, other possible applications of neurofilaments as a biological marker for ICU-AW warrant future studies. Raised neurofilaments may aid in solving differential diagnostic difficulties when differentiating between CIM, CIP or CINM, which is currently dependent on technically challenging electrophysiological studies and/or invasive muscle biopsies [2]. Additionally, raised neurofilaments could have prognostic consequences. Both axonal damage instead of dysfunction and axonal involvement instead of muscle involvement are associated with adverse outcomes in patients with ICU-AW [16, 27, 28]. However, two technical issues remain to be solved before launching new studies. Firstly, intra-laboratory CV for the neurofilament heavy chain ELISA needs to be investigated, because this was shown to be poor for neurofilament light chain ELISA [29]. Related to this, neurofilament levels obtained in this study should be interpreted with caution when implemented in other laboratories. Secondly, the reliability of the assay within the ICU environment needs to be investigated because there may be interference by blood-borne factors, such as ICU-specific medication, or by changes in plasma levels not related to axonal injury, such as dilution because of fluid resuscitation or accumulation because of decreased clearance.

## Conclusions

Clinically, there is a need for alternative diagnostic approaches that allow for early diagnosis of ICU-AW. The current approach, using manual muscle strength assessment, is frequently delayed and important prognostic information is thus withheld. While plasma neurofilament levels are raised in ICU-AW, the time at which these levels peak precludes the use of this neurofilament assay as an early diagnostic marker for ICU-AW.

## Key messages

- Plasma neurofilament levels, a biomarker for axonal damage, are raised in patients with Intensive Care Unit–acquired weakness.
- An early diagnosis of Intensive Care Unit–acquired weakness before muscle strength assessment is probably not possible using plasma neurofilament levels.

## Electronic supplementary material

Additional file 1: Figure S1: Neurofilaments in patients with and without Intensive Care Unit-acquired weakness and in patients without muscle strength assessment. Neurofilament levels in all plasma samples (**A**) and peak (**B**) neurofilament levels per patients. Horizontal bars show median group values. In the no MRC group, we identified two patients with higher neurofilament levels than others. One of those patients suffered from coma due to a hepatic encephalopathy (samples denoted with +), while the other patient had prolonged delirium (samples denoted with a white square). (PDF 367 KB)

Additional file 2: Figure S2: **A**) Admission neurofilament levels for chronic neurological co-morbidities. Admission neurofilament levels (ICU day 0 or 1) for patients with and without chronic neurological co-morbidities. Horizontal black bars show median group values. Data are missing for seven patients because admission blood samples were not obtained or could not be successfully analyzed. **B**) Peak neurofilament levels for chronic neurological co-morbidities. Peak neurofilament levels for patients with and without chronic neurological co-morbidities. Horizontal black bars show median group values. (PDF 667 KB)

## Abbreviations

APACHE IV: Acute Physiology and Chronic Health Evaluation IV
AUC: area under the receiver operating characteristic curve
CIM: critical illness myopathy
CINM: critical illness neuromyopathy
CIP: critical illness polyneuropathy
CNS: central nervous system
CV: coefficient of variation
ELISA: enzyme-linked immunosorbent assay
ICU: intensive care unit
ICU-AW: intensive care unit–acquired weakness
IQR: interquartile range
MODS: multiple organ dysfunction syndrome
MRC: Medical Research Council
RASS: Richmond Sedation and Agitation Scale
ROC: receiver operating characteristic curve
SOFA: Sequential Organ Failure Assessment
STARD: Standards for the Reporting of Diagnostic accuracy studies.
