# Supplementary material for: Neurofilaments as a plasma biomarker for ICU-acquired weakness: an observational pilot study
Source: Crit Care. 2014 Jan 20;18(1):R18. doi: 10.1186/cc13699 (PMC4057240; doi:10.1186/cc13699)
Supplement: Supplementary file 1 — Additional file 1: Figure S1: Neurofilaments in patients with and without Intensive Care Unit-acquired weakness and in patients without muscle strength assessment. Neurofilament levels in all plasma samples (A) and peak (B) neurofilament levels per patients. Horizontal bars show median group values. In the no MRC group, we identified two patients with higher neurofilament levels than others. One of those patients suffered from coma due to a hepatic encephalopathy (samples denoted with +), while the other patient had prolonged delirium (samples denoted with a white square). (PDF 367 KB) [file 13054_2013_2856_MOESM1_ESM.pdf]

Additional file 1: Figure S1.

Neurofilaments in patients with and without Intensive Care Unit-acquired weakness and in patients without muscle strength assessment

Neurofilament levels in all plasma samples (panel A) and peak (panel B) neurofilament levels per patients. Horizontal bars show median group values. In the no MRC group, we identified two patients with higher neurofilament levels than others. One of those patients suffered from coma due to a hepatic encephalopathy (samples denoted with +), while the other patient had prolonged delirium (samples denoted with □).

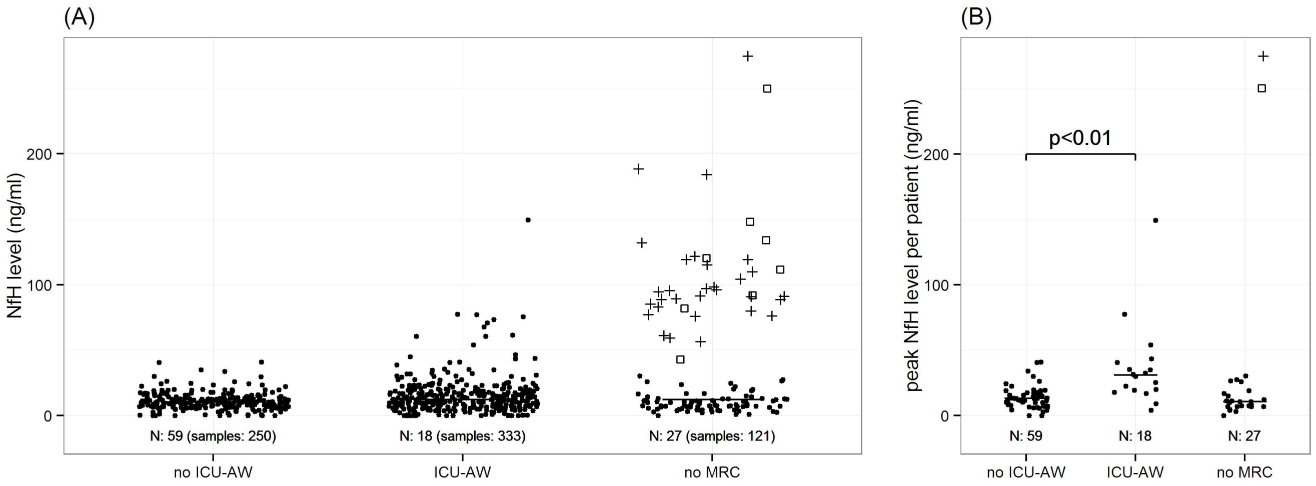

NfH: neurofilaments; MRC: Medical Research Council; ICU-AW: Intensive Care Unit – acquired weakness
