# Supplementary material for: Neurofilaments as a plasma biomarker for ICU-acquired weakness: an observational pilot study
Source: Crit Care. 2014 Jan 20;18(1):R18. doi: 10.1186/cc13699 (PMC4057240; doi:10.1186/cc13699)
Supplement: Supplementary file 2 — Additional file 2: Figure S2: A) Admission neurofilament levels for chronic neurological co-morbidities. Admission neurofilament levels (ICU day 0 or 1) for patients with and without chronic neurological co-morbidities. Horizontal black bars show median group values. Data are missing for seven patients because admission blood samples were not obtained or could not be successfully analyzed. B) Peak neurofilament levels for chronic neurological co-morbidities. Peak neurofilament levels for patients with and without chronic neurological co-morbidities. Horizontal black bars show median group values. (PDF 667 KB) [file 13054_2013_2856_MOESM2_ESM.pdf]

Additional file 2: Figure 2.

A) Admission neurofilament levels for chronic neurological co-morbidities

Admission neurofilament levels (ICU day 0 or 1) for patients with and without chronic neurological co-morbidities. Horizontal black bars show median group values. Data missing for 7 patients because admission blood samples were not obtained or could not be successfully analyzed.

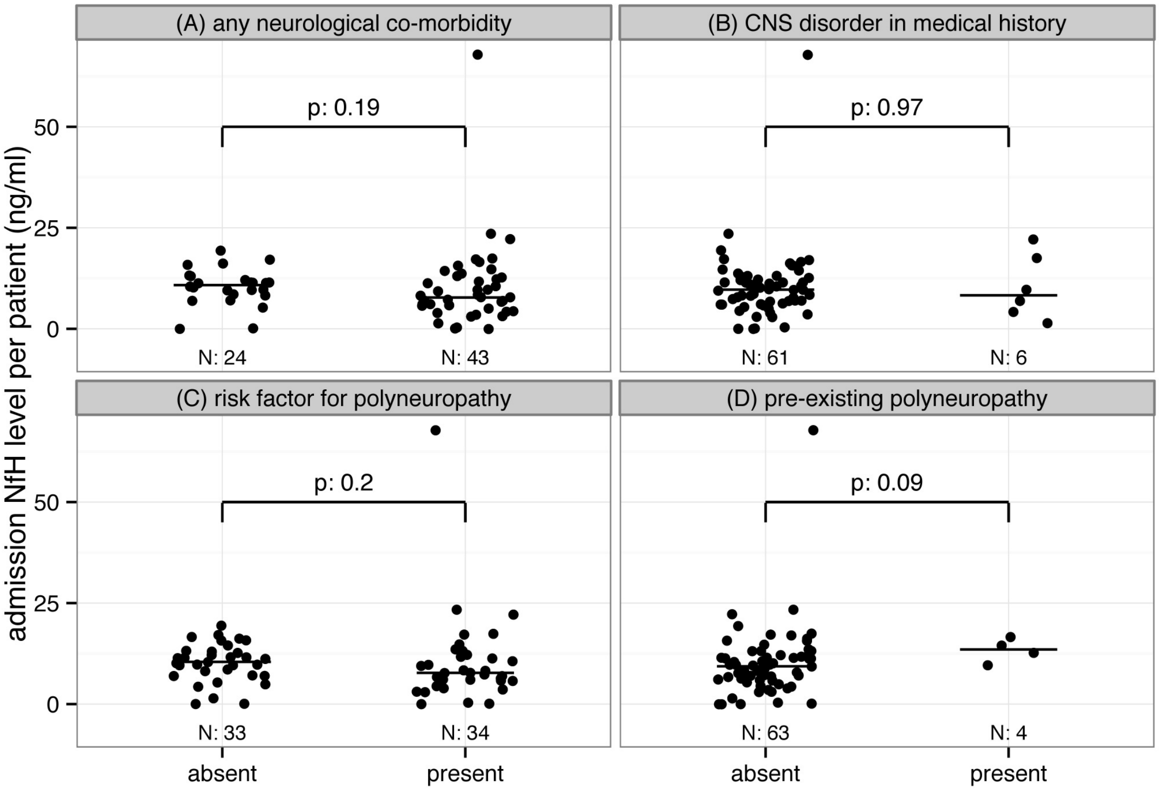

NfH: neurofilaments; CNS: central nervous system

B) Peak neurofilament levels for chronic neurological co-morbidities

Peak neurofilament levels for patients with and without chronic neurological co-morbidities. Horizontal black bars show median group values.

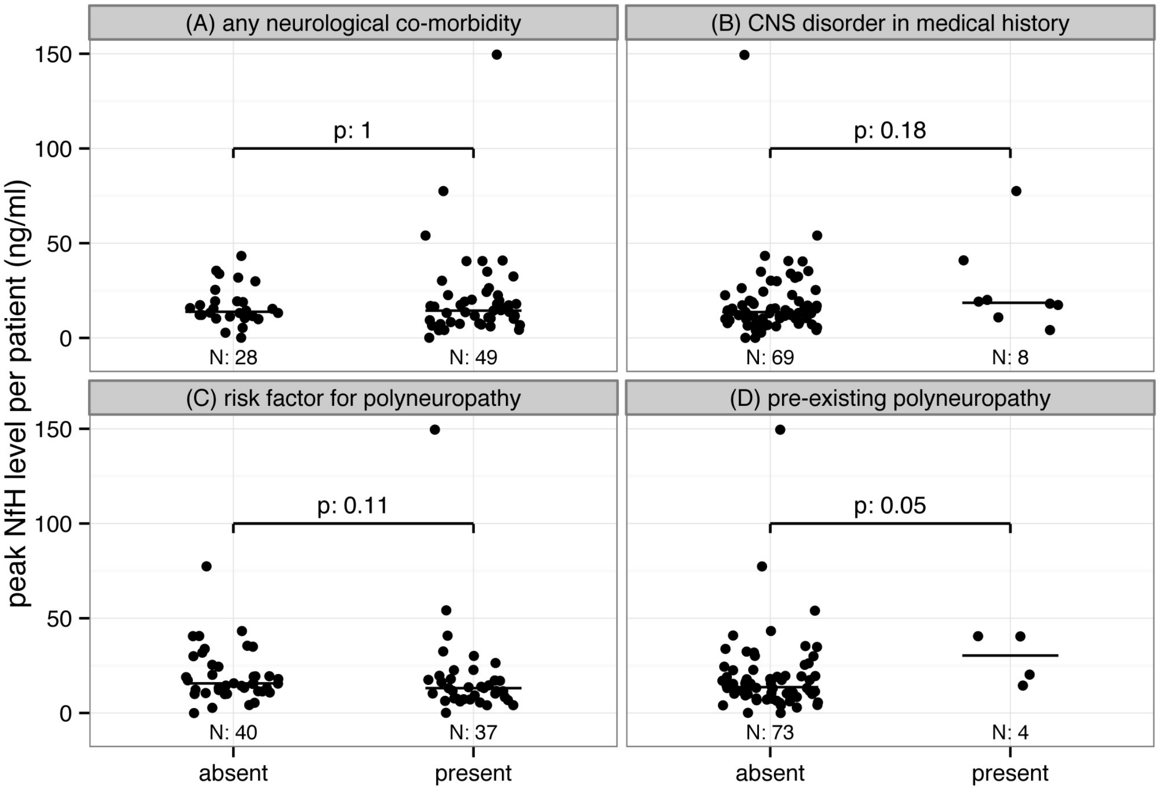

NfH: neurofilaments; CNS: central nervous system
